# AutoMap is a high performance homozygosity mapping tool using next-generation sequencing data

**DOI:** 10.1038/s41467-020-20584-4

**Published:** 2021-01-22

**Authors:** Mathieu Quinodoz, Virginie G. Peter, Nicola Bedoni, Béryl Royer Bertrand, Katarina Cisarova, Arash Salmaninejad, Neda Sepahi, Raquel Rodrigues, Mehran Piran, Majid Mojarrad, Alireza Pasdar, Ali Ghanbari Asad, Ana Berta Sousa, Luisa Coutinho Santos, Andrea Superti-Furga, Carlo Rivolta

**Affiliations:** 1 Institute of Molecular and Clinical Ophthalmology Basel (IOB), Basel, Switzerland; 2 Department of Ophthalmology, University of Basel, Basel, Switzerland; 3 Department of Genetics and Genome Biology, University of Leicester, Leicester, UK; 4 Institute of Experimental Pathology, Lausanne University Hospital (CHUV), Lausanne, Switzerland; 5 Service of Medical Genetics, Lausanne University Hospital (CHUV), Lausanne, Switzerland; 6 Department of Medical Genetics, Faculty of Medicine, Mashhad University of Medical Sciences, Mashhad, Iran; 7 Noncommunicable Diseases Research Center, Fasa University of Sciences, Fasa, Iran; 8 Department of Medical Genetics, Hospital Santa Maria, Centro Hospitalar Universitário Lisboa Norte (CHULN), Lisbon Academic Medical Center (CAML), Lisbon, Portugal; 9 Bioinformatics and Computational Biology Research Center, Shiraz University of Medical Sciences, Shiraz, Iran; 10 Division of Applied Medicine, Medical School, University of Aberdeen, Aberdeen, UK; 11 Medical Faculty, Lisbon University, Lisbon, Portugal; 12 Instituto de Oftalmologia Dr Gama Pinto, Lisbon, Portugal

**Keywords:** Bioinformatics, Consanguinity, Genomics, Next-generation sequencing

## Abstract

Homozygosity mapping is a powerful method for identifying mutations in patients with recessive conditions, especially in consanguineous families or isolated populations. Historically, it has been used in conjunction with genotypes from highly polymorphic markers, such as DNA microsatellites or common SNPs. Traditional software performs rather poorly with data from Whole Exome Sequencing (WES) and Whole Genome Sequencing (WGS), which are now extensively used in medical genetics. We develop AutoMap, a tool that is both web-based or downloadable, to allow performing homozygosity mapping directly on VCF (Variant Call Format) calls from WES or WGS projects. Following a training step on WES data from 26 consanguineous families and a validation procedure on a matched cohort, our method shows higher overall performances when compared with eight existing tools. Most importantly, when tested on real cases with negative molecular diagnosis from an internal set, AutoMap detects three gene-disease and multiple variant-disease associations that were previously unrecognized, projecting clear benefits for both molecular diagnosis and research activities in medical genetics.

## Abstract (web summary)

Homozygosity mapping is a useful tool for identifying candidate mutations in recessive conditions, however application to next generation sequencing data has been sub-optimal. Here, the authors present AutoMap, which efficiently identifies runs of homozygosity in whole exome/genome sequencing data.

## Introduction

Homozygosity mapping (HM), also called autozygosity mapping, is a technique aimed at detecting and scoring the presence of consecutive homozygous genotypes, or “runs of homozygosity” (ROHs) in a person’s genome. ROHs result from the co-inheritance of portions of DNA that are prevalent in a given population, and normally range from a few to hundreds of megabases (Mb), depending on the ethnic group considered^1,2^. In the offspring of consanguineous unions, ROHs correspond in large part to the genetic material that is co-inherited from ancestors who are common to both parents^3^. For instance, children of parents who are first cousins have ROHs for DNA regions that were in heterozygosis in their great-grandparents, and were inherited from both their maternal and paternal sides.

In patients with recessive conditions, especially if belonging to a consanguineous pedigree, ROHs very often encompass the mutation that is responsible for the disease^4^, also originating from a healthy heterozygous common ancestor, and therefore are excellent proxy markers for the mutation itself. In addition, HM can also be used to detect large heterozygous deletions resulting in hemizygous (and apparently homozygous) genotypes, as well as for cases of uniparental disomy^5,6^. For these reasons, HM has been used for decades in medical genetics as a tool to identify regions of the genome to be prioritized for targeted mutational screens. In the past, HM procedures relied on genotypes from polymorphic markers (typically microsatellites) to determine whether patients had consecutive homozygous calls vs. healthy relatives. Since the beginning of this century, the commercialization of high-density microarrays for single-nucleotide polymorphisms (SNPs) has allowed the use of the SNP genotypes for the same purpose, by interrogating usually non-coding variants with an elevated degree of heterozygosity in the general population. Several software were then developed, such as PLINK^7^, HomozygosityMapper^8^, or GERMLINE^9^. In virtue of the very high accuracy of their calls (>99.8%)^10^, SNP microarrays outputs are highly reliable to identify ROHs. However, they do not provide any information on a patient’s mutations, since these latter DNA variants are usually rare and therefore are not included in such arrays.

Conversely, information from whole-exome sequencing (WES), which is largely used in contemporary medical genetics, allows the discovery of any type of DNA variants, including frequent genotypes and rare mutations alike. Therefore, at least in principle, WES can be used as a single technology for both HM and mutation detection. However, HM algorithms developed for array technologies tend to deliver sub-optimal results when applied to WES, since they are not adapted to handle the intrinsic noise (2.53–30.60%)^10^ that is typical of this sequencing method^11–13^. In particular, ROHs detected from WES data with this software are smaller in size and produce lower cumulative autozygosity values, often resulting in the end in falsely-negative output^14–16^. Hence, many investigators still make use of two separate tools: SNP microarrays to determine ROHs, and WES to identify possible rare mutations, with an important waste of time and resources^17–21^.

To circumvent this problem, in this work we present AutoMap (Autozygosity Mapper), a software that can provide very reliable HM results directly from standard WES outputs and is applicable to WGS (whole-genome sequencing) as an additional feature, and compare it with other tools, including some that were specifically developed to perform HM on WES data (BCFTools^16^, FILTUS^22^, H^3^M^2^^23^, HOMWES^15^, SavvyVcfHomozygosity, and SavvyHomozygosity^24^). Each of the tools used in this comparison have their own strongpoints and limitations, such as the presence of a graphical user interface (GUI), a cross-platform operative system, a user-friendly output type, etc^25,26^. AutoMap is accessible both via a web interface (free of charge), to allow low-throughput or occasional use for exome data, and as a command-line tool, for large-scale genomic projects, for WGS, and to allow integration into routine analytical pipelines.

## Results and discussion

**Fig. 1:**
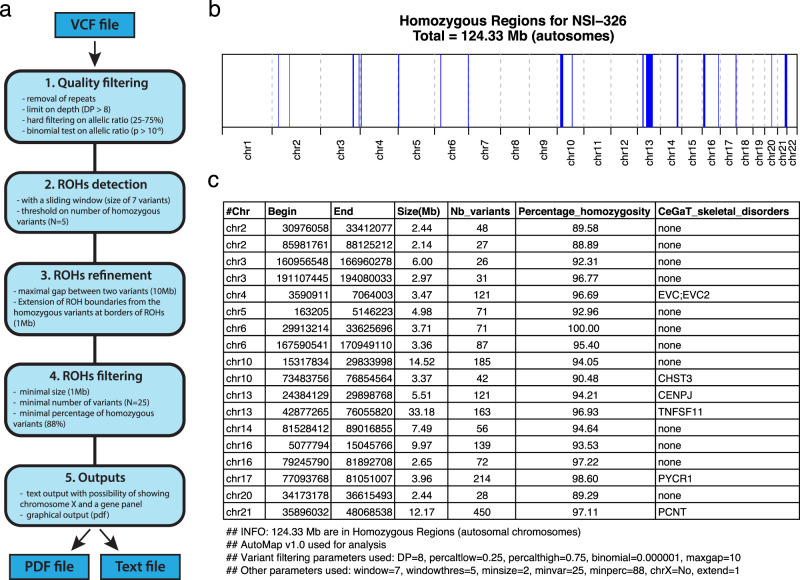
AutoMap workflow and example of output. **a** Workflow followed by AutoMap with default settings, **b**, **c** example of graphical and text output for patient NSI-326, with the following parameters: DP = 8, percaltlow = 0.25, percalthigh = 0.75, binomial = 0.000001, maxgap = 10, window = 7, windowthres = 5, minsize = 2, minvar = 25, minperc = 88, chrX = No, and extend = 1. Blue regions represent detected ROHs.

AutoMap takes as an input variant call format (VCF) files (Fig. 1a), i.e., standard result files from a variety of commonly-used software for analyzing WES and WGS sequences (variant callers), containing the list of DNA variants detected in a given sample with respect to the human reference genome. VCF files have the advantage of being of smaller size compared to primary mapping files (BAM files), contain most of the information that can be used for HM purposes and, importantly, are generally more available to the end-users, such as biologists or physicians. Of note, single-sample VCFs are accepted by both web and standalone versions of AutoMap, while multisample VCFs are only accepted by the standalone version. Once the file is uploaded via the web interface (https://automap.iob.ch/) the user simply launches the analysis, which in the end produces an output such as the one indicated in Fig. 1b, c. This includes a PDF file showing the graphical representation of autozygous regions along the autosomes, as well as a text file reporting the same information as numerical values, with the positions of the detected ROHs, their size, number of variants, and percentage of homozygosity. AutoMap can also include the X chromosome, if requested by the user, for both females and males. In females, this chromosome is treated as an autosome, whereas in males all hemizygous calls are considered as homozygous. In addition, it is possible to provide a list of genes (or a “gene panel”, e.g., a list of genes linked to a given condition) as a text file, to immediately recognize whether they are or not within a given ROH (Fig. 1c). The same procedure can also be performed on a local computer, by directly downloading the source files (https://github.com/mquinodo/AutoMap). In its standalone version, AutoMap does not need compiling but requires the installation of some additional software (BCFTools, v1.9 or later; BEDTools, v2.25.0 or later; Perl, v5.22.0 or later; R, v3.2.0 or later).

As mentioned, one of the key points for the proper detection of ROH from WES data is the targeted removal of false positive variant calls. These calls not only act as a noise with respect to true signals, but also actively lead to an artificial fragmentation of ROHs (when a heterozygous false positive call is present in a truly autozygous region) or even to the complete miss of a ROH (for regions with a low number of calls). To this end, as a first step, all variants from the VCF file are carefully assessed with criteria relying on coverage and alternative reads count. More precisely, they are evaluated according to their sequencing depth, the ratio between reads aligned to the reference sequence vs. alternative alleles (for heterozygous calls), and to their location in repeated regions of the genome (Fig. 1a and Supplementary Table 1). Variants that do not satisfy these stringent criteria are selectively and individually eliminated from further analyses. Importantly, all such criteria were verified not to be specific of any given variant calling software, with the aim of enabling analyses of VCF files produced by various programs.

After this filtering step at the variant level, ROHs are identified by a sliding-window approach, associated with four tunable parameters: the size of the window, the minimal number of homozygous variants in the window, the maximal gap between two consecutive variants in one ROH, and the maximal size of extension from the boundaries of stretches of detected ROHs (Fig. 1a and Supplementary Table 1). Following their identification, ROHs are then selected only if they reach a minimal value in terms of size, number of variants and percentage of homozygous genotypes in the region (Fig. 1a and Supplementary Table 1).

To evaluate the performances of AutoMap, we gathered data from 52 families with recessive hereditary blindness, for which we also had genotypes from both WES and SNP arrays, and selected one patient per family. These individuals were all from consanguineous pedigrees living in Portugal or in Iran, i.e., in countries with a low and an elevated degree of consanguinity at the level of the general population, respectively^27,28^. More precisely, they all had a median cumulative ROH size of 234.8 Mb (detected by PLINK on SNP array data), ranging between 90.6 and 819.5 Mb. We then split these individuals into two matched sets of 26 persons each, thus defining a training cohort and a matched validation cohort (Supplementary Data 1). As a reference for true positive values, we adopted the method described by Kancheva et al.^15^, consisting in the use of the PLINK software^7^ applied with default parameters on data from SNP arrays.

We optimized the parameters of AutoMap on the training cohort (Supplementary Table 1 and Supplementary Fig. 1A, B, see “Methods” section), and then used the optimized values to analyze the validation cohort. We performed stability analyses by varying each parameter individually, which resulted in small variation of the performances, indicative of the robustness of the method (Supplementary Fig. 1C). In addition, the average performance was not significantly different between the two cohorts, demonstrating no over-fitting of the parameters on the training data (Supplementary Fig. 2A). More precisely, specificity in the training and validation sets had values of 77.5% and 79.6%, respectively (*p* = 0.35, unpaired *t*-test), while sensitivity had values of 90.5% and 92.4%, respectively (*p* = 0.18).

We also compared the effect of three mainstream variant callers that could be adopted by AutoMap end users (GATK-Haplotype Caller, Samtools-mpileup and Strelka) on the performance of our tool (based on data from the training set). AutoMap’s sensitivity was slightly lower when Strelka was used (−1.4% compared to Haplotype Caller), whereas specificity appeared to be slightly lower when Samtools was used, (−4.4% compared to Haplotype Caller, Supplementary Fig. 2B). These differences were all below 5%, therefore indicating that our tool is overall insensitive to the choice operated by a potential user with respect to a given calling software.

Next, we compared AutoMap with eight previously-published tools, namely: PLINK applied on exome data^7^, HomozygosityMapper for WES^29^, HOMWES^15^, BCFTools/RoH^16^, FILTUS^22^, H^3^M^2^ ^23^, SavvyHomozygosity, and SavvyVcfHomozygosity^24^ on data from the validation cohort, composed of 26 individuals with various levels of autozygosity (Supplementary Fig. 3). All of them were based on a hidden Markov model with the exception of HomozygosityMapper, PLINK, and HOMWES, which were developed by applying a sliding window method (as it is the case for AutoMap as well). Of note, H^3^M^2^ and SavvyHomozygosity cannot process VCF files but need a BAM file (sequence alignment data), HomozygosityMapper is only available via a web interface, and FILTUS can only be queried via a graphical user interface (GUI), not allowing automated processing of multiple files. The use SavvyHomozygosity and SavvyVcfHomozygosity requires the downloading of pre-prepared linkage data from 1000 Genomes project or a joint vcf from a few hundred WGS. We did not include other software packages such as Agile-Genotyper, Agile-VariantMapper^14^, and HomSI^30^ since they do not provide a genome-wide result file, which could be used as source of data for comparison, but only a graphical output. We used default parameters for all these tools (i.e., the parameters optimized by their respective authors to analyze WES data), except for PLINK, since this software was first developed for SNP array data and therefore had to be re-parametrized to allow its use on WES genotypes (see “Methods” section).

**Fig. 2:**
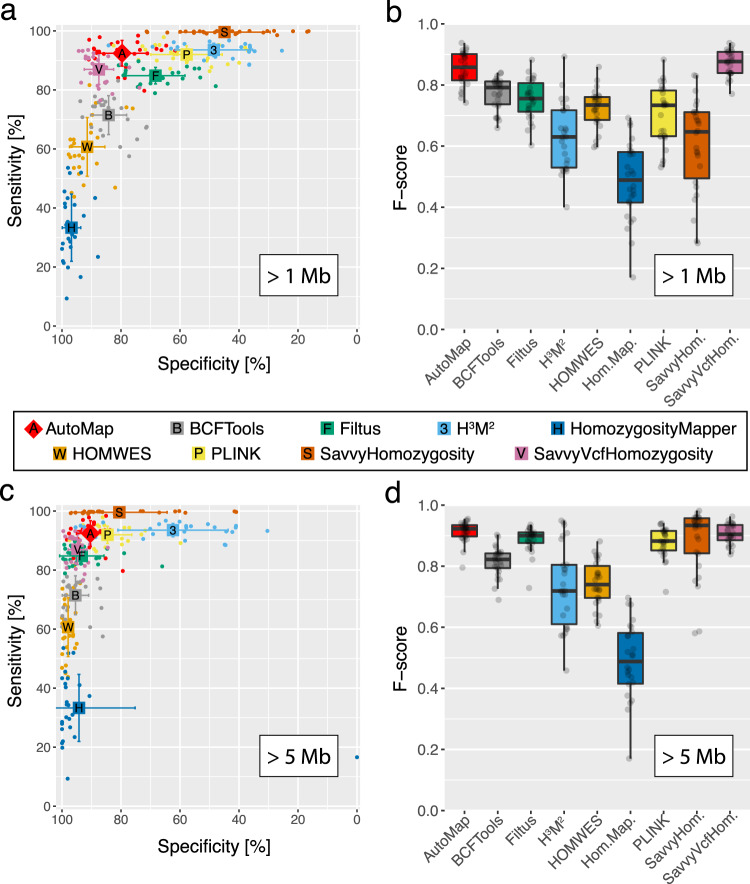
Performance of AutoMap and other tools on data from the validation set. **a**, **b** Performance of AutoMap for ROHs larger than 1 Mb (Megabase): **a** specificity and sensitivity; **b** *F*-score. **c**, **d** Same analyses as in **a**, **b**, but limited to ROHs with sizes of 5 Mb or higher (most likely to contain causative recessive mutations in medical genetics practice, according to published literature). Error bars represent standard deviations of the mean. For boxplots (**b**, **d**), the middle band indicates the median, boxes represent the first and the third quartiles, and whiskers indicate the largest observation smaller than or equal to the first quartile −1.5 x IQR (the interquartile range) and the smallest observation greater than or equal to to third quartile +1.5 x IQR. *N* = ~2.7 million DNA variants per tool and per test. Source data are provided in the Source Data File.

AutoMap and SavvyVcfHomozygosity had the best sensitivity/specificity combination and the highest *F*-score when compared to other software (*p* < 3.0 × 10^−7^, paired *t*-test) (Fig. 2a, b). However, AutoMap had a significantly higher sensitivity than SavvyVcfHomozygosity (*p* < 2.9 × 10^−6^) and lower specificity (*p* < 2.8 × 10^−6^).

**Fig. 3:**
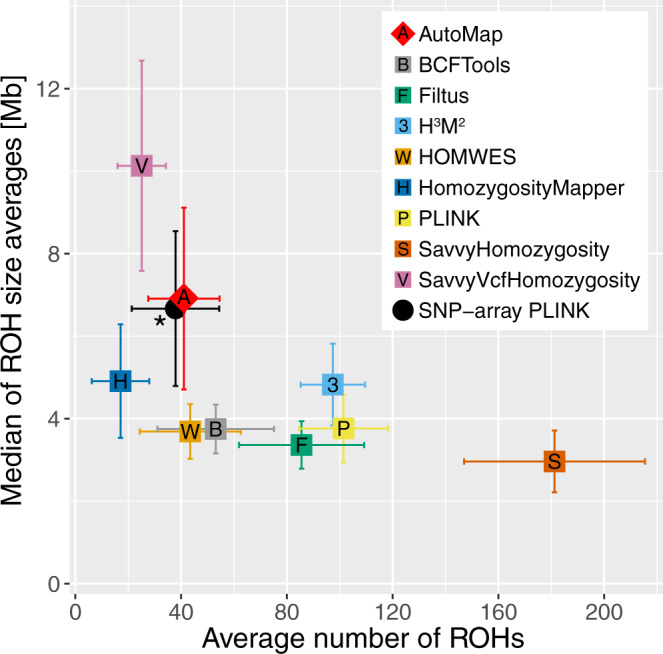
Average number of ROH detected per sample (*N* = 26) vs. the median of the size averages of ROHs per sample (ROHs larger than 1 Mb). The asterisk indicates the reference value used for comparison, i.e., data from SNP arrays analyzed with PLINK. Error bars represent standard deviations of the mean. Source data are provided in the Source Data File.

In addition, AutoMap (on WES) was the tool that displayed the closest values, in terms of ROHs size and number, to the reference (i.e., PLINK on SNP array) (Fig. 3). This indicated no or very low ROH fragmentation, especially in comparison to other tools, which produced for instance either a high number of small and fragmented ROHs (H^3^M^2^, FILTUS, and PLINK) or detected only large ROHs (SavvyVcfHomozygosity). Two representative examples of this effect, over entire choromosomes, are presented in Supplementary Fig. 4.

Since small ROHs are more difficult to detect and indeed could penalize the global performance of some of these tools, we repeated the same analysis by considering only ROHs larger than 5 Mb, being aware that such regions are more likely to harbor causative variants for recessive diseases^4^. As expected, every tool displayed increased performances, due to the artificial clearing of smaller ROHs, and AutoMap, SavvyHomozygosity and SavvyVcfHomozygosity had the best *F*-scores (*p* < 2.0 × 10^−4^, Fig. 2c, d). AutoMap had a significant higher sensitivity than SavvyVcfHomozygosity (*p* = 2.9 × 10^−6^) but lower than SavvyHomozygosity (*p* < 6.9 × 10^−9^). Concerning specificity, the results were reversed, with AutoMap having a significantly higher value than SavvyHomozygosity (*p* < 3.0 × 10^−3^) but a lower score with respect to SavvyVcfHomozygosity (*p* < 2.5 × 10^−5^). In summary, AutoMap had the best performances overall when tested on our validation cohort. Since this cohort comprised only individuals of European and South Asian ancestry, further assessments may be needed to evaluate population-specific performances on data from other ethnic groups. In addition, a possible confounding factor in our analysis could be represented by the use of different capture kits for WES. Although we did not assess this parameter at the experimental level, previous literature has shown that concordance rates of called variants across different capture kits are very elevated^31^, making substantial variability of performance linked to the use of different kits a rather unlikely event. Concerning WGS data, additional tests will be also needed to benchmark existing tools such as PLINK or other array-specific software vs. AutoMap, due to the difference in terms of number of variants and coverage compared to WES.

In addition to an increased performance in retrieving ROHs from exome data, AutoMap also displayed other benefits. For instance, AutoMap was the only tool that could be run both via a web application (for the average user) and the command-line, to allow more computationally-inclined users to exploit its full potential. In addition, the average processing time displayed by AutoMap was relatively short, especially in relationship to its performance. All tools, except those using BAM files as an input (H^3^M^2^ and SavvyHomozygosity) could analyze a standard VCF in 30 s or less. The fastest tools were BCFTools and PLINK, completing the analysis in less than 5 s, while AutoMap took approximately 20 s. A more detailed comparison of all these features is provided in Supplementary Table 2.

As a final test, we used AutoMap on unexplored WGS and WES research data from our laboratory, for cases with unknown molecular etiology. Following experimental validation, our tool was instrumental in the end for identifying both coding and noncoding mutations, all included into larger ROHs, for a few new syndromes. These included: an intergenic deletion causing developmental defects^32^, a small deletion affecting splicing in the gene *PISD* and resulting in the Liberfarb syndrome^33^, and a partial duplication of the *NMNAT1* gene, responsible for a new multisystem disorder^34^. Compared to the other tools analyzed, AutoMap was the only one allowing the complete detection of the autozygous regions containing the mutations in all three cases (Supplementary Table 3). For the *NMNAT1* study, for example, the lack of performance displayed by some other tools was likely due to the presence of a few false-positive heterozygous variants in the ROH containing the pathogenic duplication, which were indeed recognized as such—and hence discarded—by AutoMap. The same was true for the two other studies, where the presence of false-positive heterozygous genotypes was combined with the relatively short size of ROHs and/or the presence of a limited number of identifiable genotypes. In addition, AutoMap enabled the detection of new variants in a number of genes that were already associated with Mendelian conditions^35–37^. In conclusion, AutoMap is a reliable tool that can predict ROHs with high specificity and sensitivity, in less than a minute, even from VCF files derived from noisy exome sequencing data. It is available both via a web-based interface, for a quick analysis, as well as a command-line package, allowing large-scale and routine analyses.

## Methods

### Patients and DNA

This study adhered to the to the tenets of the Declaration of Helsinki and was approved by the Institutional Review Boards of our respective institutions: the Ethikkommission Nordewest- and Zentralschweiz (2019-01660), the Institutional Review Boards of Mashhad University of Medical Sciences (961015), the ethics commission of the Ophthalmic Hospital “Dr Gama Pinto” in Lisbon (16.05.20) and the Noncommunicable Diseases Research Center of Fasa University of medical sciences (IR.FUMS.REC.1396.211). Written informed consent forms were signed by all subjects, recruited at the Ophthalmic Hospital “Dr Gama Pinto” in Lisbon, and at the Fasa and Mashhad Universities of Medical Science in Iran. Participants had either reported consanguinity or had ~100 Mb or more of cumulative ROHs as determined by PLINK applied on array data (Supplementary Data 1). Genomic DNA was extracted from peripheral blood leukocytes. More precisely, for Portuguese patients DNA extraction was performed by using the EZ1 DNA blood kit and EZ1 DNA buffy coat card (Qiagen), according to the manufacturer’s instructions, choosing an elution volume of 200 μl. For Iranian patients, DNA was extracted from blood using RPN8512 Nucleon BACC3 DNA Extraction Kit (Illustra).

### Array genotyping

DNA of studied individuals were genotyped at the iGE3 Platform of the University of Geneva, Switzerland, using Illumina Infinium arrays (San Diego, USA; GSAMD-24v2.0, GSA-24v2.0, CoreExome-24v1.1, and CoreExome-24v1.2). Genotypes values were obtained with GenomeStudio (Illumina).

### Exome sequencing

Exome capture and library preparation was performed using the SureSelect Human All Exon v6 kit (Agilent, SantaClara, USA) and HiSeq Rapid PE Cluster Kit v2 (Illumina, San Diego, USA) with 2 μg genomic DNA. Libraries were sequenced on a HiSeq 2500 or a NovaSeq 6000 instruments (Illumina) (Supplementary Data 1). Raw reads were mapped to the human genome reference sequence (build hg19) with the Novoalign software (V3.08.00, Novocraft Technologies, Selangor, Malaysia). Duplicate reads were then removed using Picard (v. 2.14.0-SNAPSHOT). Base quality score recalibration was performed and variant calling was done with HaplotypeCaller (GATK, v.4.0.3.0).

### AutoMap requirements

AutoMap is composed of Bash, Perl and R scripts. It requires BCFTools (≥v1.9), BEDTools (≥v2.25.0), Perl (≥v5.22.0), and R (≥v3.2.0). The following versions were used for all analysis: BCFTools (v1.9-78-gb7e4ba9), BEDTools (v2.25.0), Perl (v5.22.0), Bash (4.3.48(1)-release) and R (v3.5.1).

To be processed by AutoMap, a VCF file must contain the GT (genotype) and AD (allelic depths for the ref and alt alleles) or DP4 (number of high-quality ref-forward bases, ref-reverse, alt-forward, and alt-reverse bases) fields.

### AutoMap algorithm

The first step of the algorithm removes variants from the VCF file that are located in repeat regions, as reported by the UCSC genome browser (https://genome.ucsc.edu/cgi-bin/hgTables). Variants are then filtered by quality, based on the percentage of alternative reads (default options: –minpercalt 0.25 and –maxpercalt 0.75), on a binomial test for alternative and reference read counts for heterozygous variants (default option: –binomial 0.000001), and on depth (default option: –DP 8). At this point, the analysis is stopped if there are less than 10,000 variants surviving such procedures.

ROHs are subsequently detected by a sliding window with a fixed size and a threshold based on the number of homozygous variants (default options: –window 7 variants and –windowthres 5). After that, detected ROHs are trimmed at their ends to remove heterozygous variants and are extended at both ends (default option: –extend 1 [Mb]). ROHs containing regions without variants for a stretch larger than a given threshold (default option: –maxgap 10 [Mb]) are split into two different ROHs by excluding the region with no variants. Finally, ROHs are filtered to have a minimal size, a minimal number of variants and a minimal percentage of homozygous variants (default options: –minsize 1 [Mb], –minvar 25, and –minperc 88 [%]).

### Probabilities for binomial distribution

Probabilities for binomial distributions were calculated with the Perl script written by T.J. Finney (https://www.halotype.com/RKM/figures/TJF/binomial.txt).

### Overlap of ROHs

Overlap of ROHs were obtained with bedtools and the following command:

*bedtools intersect -a a.bed -b b.bed*

### Parameters used to detect ROHs from array data

PLINK was used with default parameters, except for variants with minor allele count of 2 per batch, for filtering against very rare variants, often representing false-positive^38,39^:

*plink* –*bfile bfile* –*homozyg* –*out out* –*homozyg-window-het 1* –*homozyg-density 50* –*homozyg-gap 1000* –*homozyg-window-missing 5* –*homozyg-window-snp 50* –*homozyg-snp 100* –*homozyg-window-threshold 0.05* –*homozyg-kb 1000* –*mac 2*

### Detection of ROHs from exome data

All available tools were used with default parameters for treating input files, optimized by their developers, except for PLINK. For this tool, we took the parameters defined by Kancheva et al. ^15^, to optimize sensitivity, and re-tested the most important parameter, i.e., the number of allowed heterozygous SNP per ROH. This parameter is important to take in account the noise in exome sequencing data. Indeed, low values of this parameter resulted in low sensitivity. We chose to use a value of 3 since it produced a higher sensitivity with acceptable specificity (Supplementary Fig. 5).

The command used was:

*plink* –*bfile bfile* –*homozyg* –*out out* –*homozyg-kb 1000* –*homozyg-window-het 3* –*homozyg-density 10000* –*homozyg-gap 10000* –*homozyg-window-missing 10* –*homozyg-window-snp 20* –*homozyg-snp 10* –*homozyg-window-threshold 0.05*

For SavvyHomozygosity and SavvyVcfHomozygosity, we used the pre-prepared linkage data from 1000 Genomes project, provided by the developers (https://github.com/rdemolgen/SavvySuite).

### Parameter used for variant callers

Samtools:

*samtools mpileup -t DP,AD -ugf ref.fasta input.bam*|*bcftools call -vmO v -o out.vcf*

Strelka:

*configureStrelkaGermlineWorkflow.py* –*bam input.bam* –*referenceFasta ref.fasta* –*exome*

HaplotypeCaller:

*gatk* –*java-options “-Xmx4g” HaplotypeCaller -R ref.fasta* –*dbsnp dbsnp.vcf -I $input.bam -O out.vcf*

After calling, variants were filtered to be present in the regions included in the capture kits, with a 100 bp padding on each side.

### Computations of performances

As a reference for true positive values, we adopted the method described by Kancheva et al.^15^, consisting in the use of the PLINK software^7^ applied with default parameters on data from SNP arrays. Sensitivity and specificity were then computed as follows:

$${\mathrm{Sensitivity}}\left( \% \right) = {\mathrm{ROH}}_{{\mathrm{exome-all}}}\,{\mathrm{overlapping}}\,{\mathrm{with}}\,{\mathrm{ROH}}_{{\mathrm{array-filtered}}}/{\mathrm{Total}}\,{\mathrm{ROH}}_{{\mathrm{array-filtered}}},$$

$${\mathrm{Specificity}}\,\left( \% \right) = {\mathrm{ROH}}_{{\mathrm{exome-filtered}}}\,{\mathrm{overlapping}}\,{\mathrm{with}}\,{\mathrm{ROH}}_{{\mathrm{array-all}}}/{\mathrm{Total}}\,{\mathrm{ROH}}_{{\mathrm{exome-filtered}}},$$

where filtered ROHs are larger than 1 or 5 Mb and excluding gap regions such as centromeres, telomeres, short arms, and heterochromatin defined from the gap table in UCSC Table Browser (https://genome.ucsc.edu/cgi-bin/hgTables).

*F*-score was also added as a measure of accuracy, computed as:

$${{F{-}\mathrm{score}}} = 2 * {\mathrm{sensitivity}} * {\mathrm{specificity}}/\left( {{\mathrm{sensitivity}} + {\mathrm{specificity}}} \right).$$

### Statistical tests

*T*-test were performed with t.test function in RStudio (v1.0.153) with R (v3.5.1).

For paired test: *t.test(x, y, alternative* = *“two.sided”, paired* = *TRUE)*

For unpaired test: *t.test(x, y, alternative* = *“two.sided”, paired* = *FALSE)*

### Figures

Figures were done with ggplot2 (v3.3.2) and gridExtra (v2.3) packages in RStudio (v1.0.153) with R (v3.5.1).

### Reporting summary

Further information on research design is available in the Nature Research Reporting Summary linked to this article.

## Supplementary information

Supplementary Information

Description of Additional Supplementary Files

Supplementary Data 1

Reporting Summary

## Data Availability

Array genotyping data and exome sequencing data cannot be shared because of restrictions related to the protection of personal data, as per Swiss and European law. Data from Figs. 2 and 3 can be retrieved in the Source Data file. Source data are provided with this paper.

## Source data

Source Data

## Code availability

AutoMap code is freely available on GitHub (https://github.com/mquinodo/AutoMap/) and was deposited in the zenodo.org repository (10.5281/zenodo.4279125)^40^.
