# Supplementary material for: AutoMap is a high performance homozygosity mapping tool using next-generation sequencing data
Source: Nat Commun. 2021 Jan 22;12:518. doi: 10.1038/s41467-020-20584-4 (PMC7822856; doi:10.1038/s41467-020-20584-4)
Supplement: Supplementary file 1 — Supplementary Information [file 41467_2020_20584_MOESM1_ESM.pdf]

Supplementary Information

AutoMap is a high performance homozygosity mapping tool using Next-Generation Sequencing data

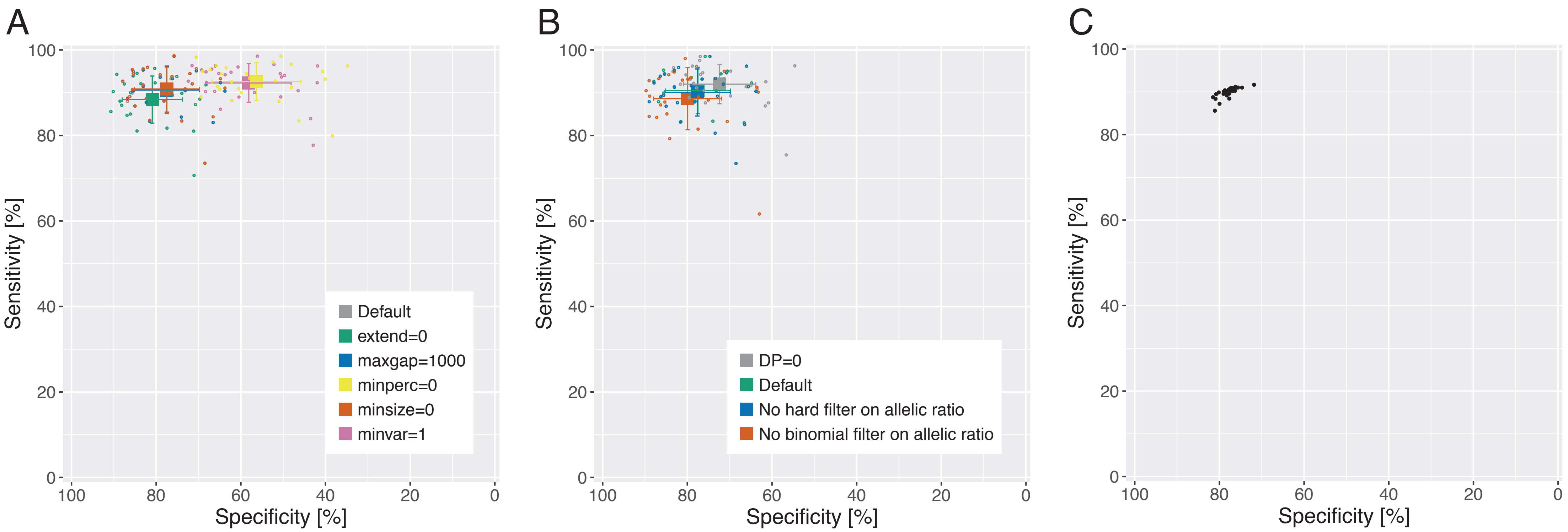

Supplementary Figure 1. AutoMap performance tests. (A) Effect of extension, maximal gap allowed, and ROHs filtering parameters (minimal percentage of homozygous variant, minimal size, and minimal number of variants) on the training set (N=26); (B) Effect of quality filtering on specificity and sensitivity of AutoMap on the training set (n=26). Error bars represent standard deviation of the mean; (C) Effect of varying single parameters on specificity and sensitivity (list of changes for all parameters can be found in the Source Data file).

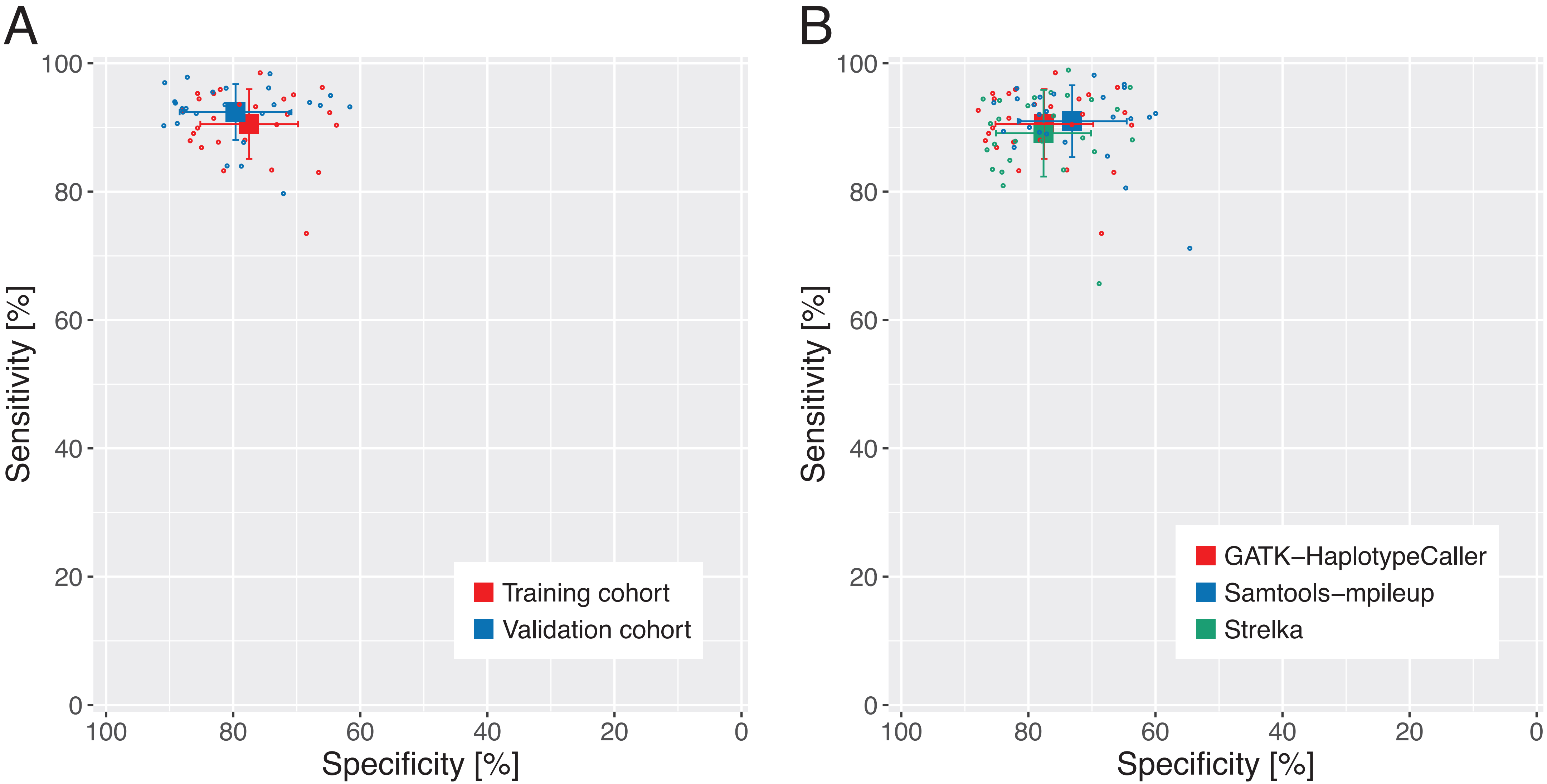

Supplementary Figure 2. Training and validation, as well as effects of variant callers. (A) Specificity and sensitivity of AutoMap with default parameters on the training (n=26) and validation (n=26) sets. (B) Sensitivity and specificity of AutoMap with default parameters for the training set (n=26), using VCF produced by different variant callers. Error bars represent standard deviation of the mean. Raw data are provided in the Source Data file.

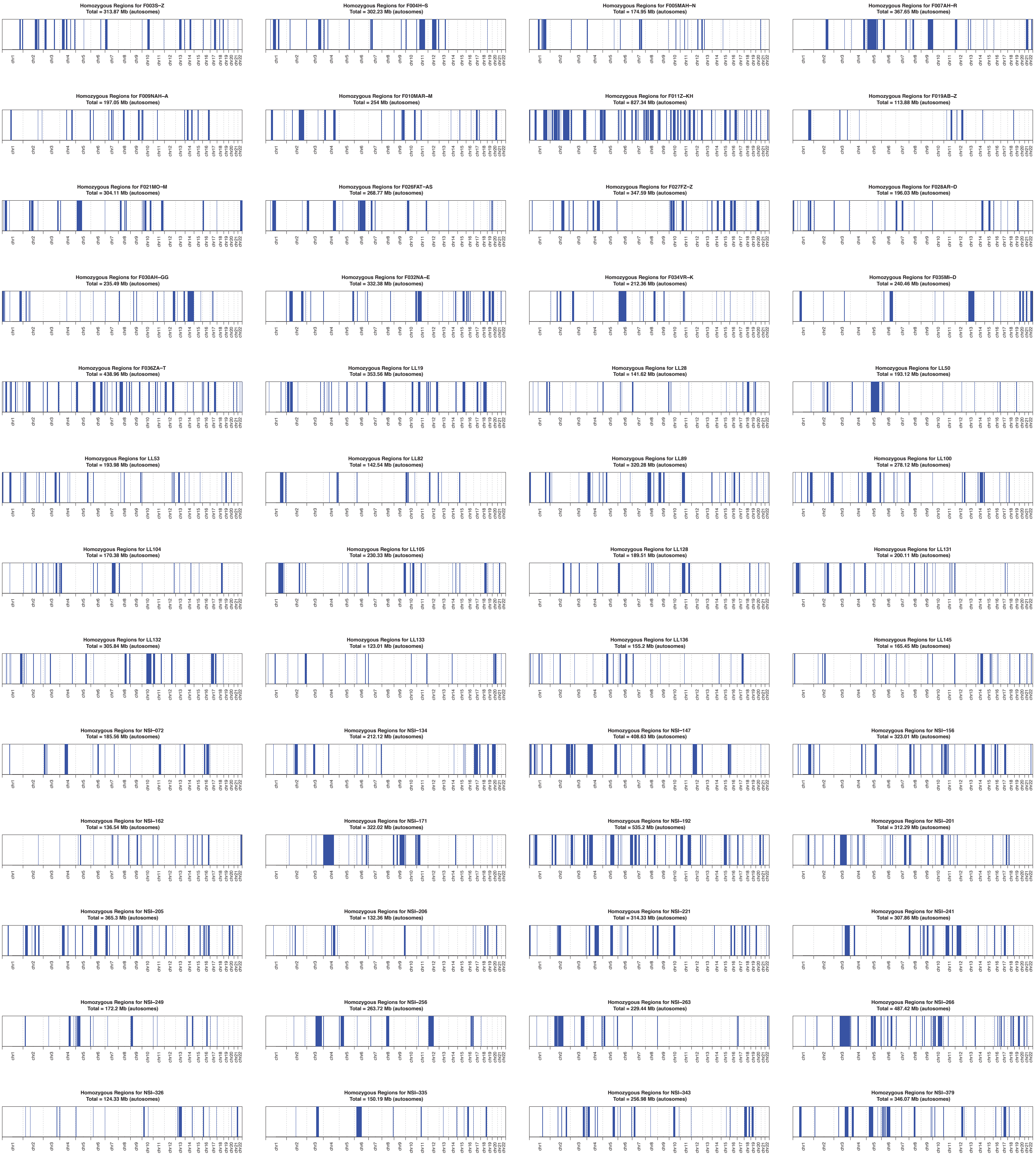

Supplementary Figure 3: Different levels of autozygosity in the 52 patients analyzed (for illustration purposes).

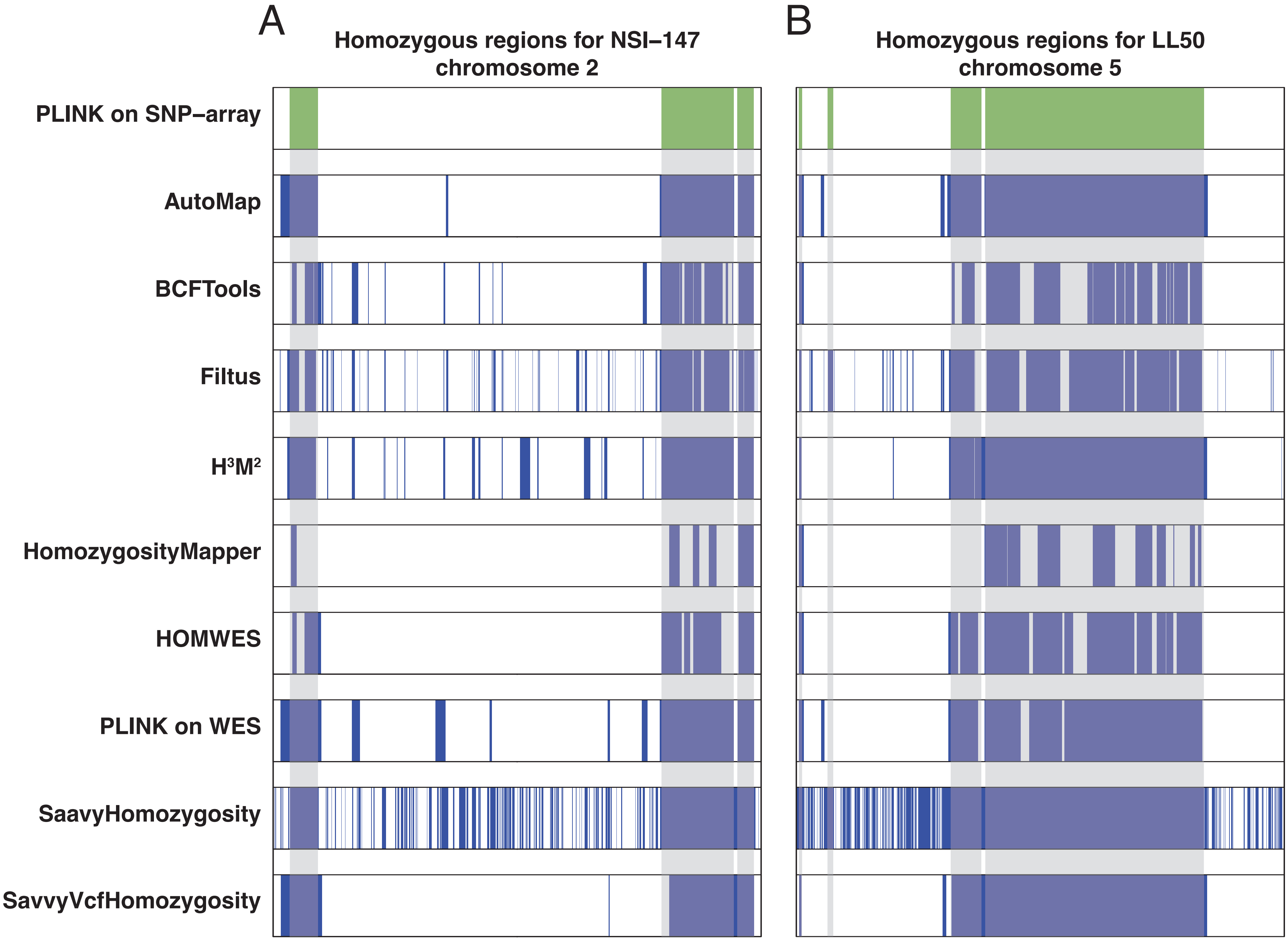

Supplementary Figure 4: Representative examples of homozygous regions found by different software, with respect to individual chromosomes. (A) Chromosome 2 for individual NSI-147; (B) Chromosome 5 for individual LL50. Grey regions represent the ROHs found by PLINK on SNP-array data (true positives).

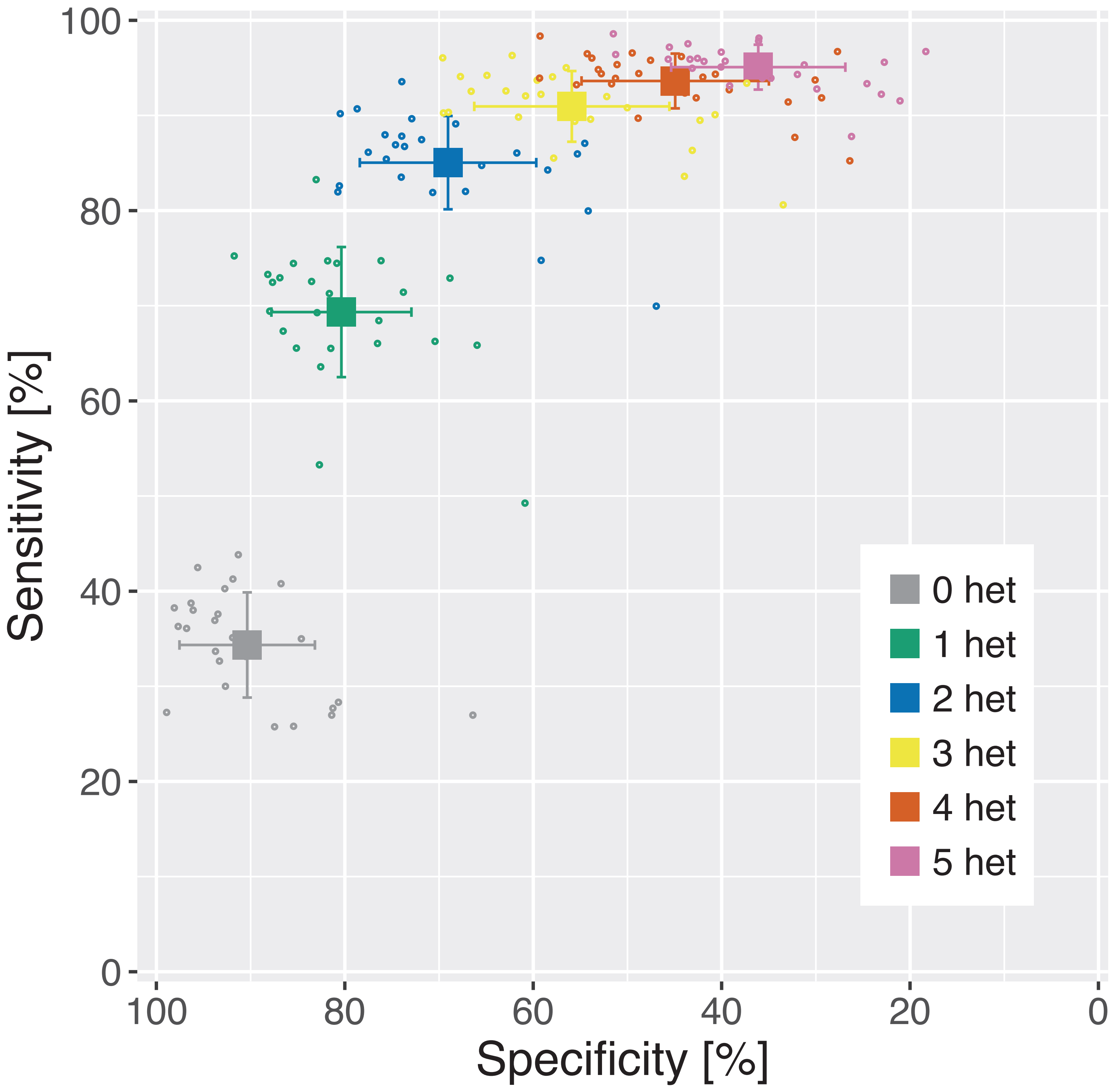

Supplementary Figure 5: Specificity and sensitivity of PLINK applied to exome data from the training set (n=26), with an incremental number of heterozygous SNP allowed per ROH (--homozyg-window-het 0 to 5), to retrieve ROHs detected by PLINK on SNP array data. Error bars represent standard deviation of the mean. Source data are provided in the Source Data file.

Supplementary Table 1. Parameters used by AutoMap, description, and possible ranges.

Mandatory options

Option	Value	Description
--vcf	String	VCF file of the individual to analyze
--genome	[hg19/hg38]	Genome build used in the VCF file
--out	String	Output directory in which the directory with the individual name will be created

Other options

Option	Value / range	Description	Default
--pat	String	Name of the individual analyzed	from VCF
--panel	String	File containg a gene or region panel (see panel format)	None
--panelname	String	Name of the panel file for output	None
--DP	0-99	Minimal depth for variants	8
--binomial	0-1	Minimal p-value for binomial test for reference and alternative alleles counts	0.000001
--percaltlow	0-1	Minimal allternative reads ratio for heterozygous variants	0.25
--percalthigh	0-1	Maximal allternative reads ratio for heterozygous variants	0.75
--window	3-999	Size of the sliding window	7
--windowthres	1-999	Threshold of homozygous variants in the window	5
--minsize	0-99	Minimal size of detected ROH [Mb]	1
--minvar	1-999	Minimal number of variant in detected ROH	25
--miperc	0-100	Minimal percentage of homozygous variants in detected ROH	88
--maxgap	0-1000	Maximal gap allowed between two variants in one ROH [Mb]	10
--extend	0-100	Maximal extension at both ROH boundaries (if no heterozygous SNPs closer)	1
--chrX	-	Outputs will contain chromosome X	No

Supplementary Table 2. Comparison of performances, computing time and user interface available for different HM tools.

Tool	Performance*	Computing time**	User interface***	Comment
AutoMap	Very good	Fast	GUI and CLI	
BCFTools	Good	Very fast	CLI only	
Filtus	Good	Fast	GUI only	
H3M2	Medium	Slow	CLI only	
HomozygosityMapper	Low	Fast	GUI only	Initially developped for SNP arrays
HOMWES	Good	Fast	CLI only	
PLINK	Good	Very fast	CLI and GUI****	Initially developped for SNP arrays
SavvyHomozygosity	Medium	Slow	CLI only	
SavvyVcfHomozygosity	Very good	Fast	CLI only	

* Based on median F-score value for >1Mb ROHs (>0.8: very good, 0.7-0.8: good, 0.6-0.7: medium, <0.6: low)

** Based on time on typical exome data on standard Linux server (<10 seconds: very fast, 10-60seconds: fast, >60seconds: slow)

*** CLI = command-line interface, GUI = graphical user interface

**** gPLINK

Supplementary Table 3. Comparison of different HM tools in the detection of mutation-containing ROHs in real datasets from patients with hereditary syndromes.

Tool	Study 1 (*EN1*)	Study 2 (*NMNAT1*)	Study 3 (*PISD*)
AutoMap	Yes	Yes	Yes
HOMWES	No	No	Yes but incomplete*
BCFTools	Yes	No	Yes but incomplete*
SavvyVcfHomozygosity	No	Yes	Yes
PLINK on WES	Yes	No	Yes but incomplete*
HomozygosityMapper	Yes	No	No
Filtus	No	Yes but incomplete*	No
SavvyHomozygosity	No	Yes	Yes
H^3M^2	Yes	Yes	Yes but incomplete*

*more than 500kb missing inside the region
