# Supplementary material for: AutoMap is a high performance homozygosity mapping tool using next-generation sequencing data
Source: Nat Commun. 2021 Jan 22;12:518. doi: 10.1038/s41467-020-20584-4 (PMC7822856; doi:10.1038/s41467-020-20584-4)
Supplement: Supplementary file 2 — Description of Additional Supplementary Files [file 41467_2020_20584_MOESM2_ESM.docx]

**Description of Additional Supplementary Files**

File Name: Supplementary Data 1.

Description: IDs, gender, age, genotyping array, NGS instrument, and DNA capture kit for samples from the training and validation sets. The level of consanguinity is reported, if known. The total size of ROHs regions larger than 1Mb detected by PLINK on autosomes and various metrics on sequencing and variants are also indicated.
