# Supplementary material for: AutoMap is a high performance homozygosity mapping tool using next-generation sequencing data
Source: Nat Commun. 2021 Jan 22;12:518. doi: 10.1038/s41467-020-20584-4 (PMC7822856; doi:10.1038/s41467-020-20584-4)
Supplement: Supplementary file 4 — Reporting Summary [file 41467_2020_20584_MOESM4_ESM.pdf]

nature research

Corresponding author(s): Carlo Rivolta
Last updated by author(s): Nov 20, 2020

Reporting Summary

Nature Research wishes to improve the reproducibility of the work that we publish. This form provides structure for consistency and transparency in reporting. For further information on Nature Research policies, see Authors & Referees and the Editorial Policy Checklist .

Statistics

For all statistical analyses, confirm that the following items are present in the figure legend, table legend, main text, or Methods section.

n/a	Confirmed	
☐	☒	The exact sample size (n) for each experimental group/condition, given as a discrete number and unit of measurement
☒	☐	A statement on whether measurements were taken from distinct samples or whether the same sample was measured repeatedly
☐	☒	The statistical test(s) used AND whether they are one- or two-sided<br>*Only common tests should be described solely by name; describe more complex techniques in the Methods section.*
☒	☐	A description of all covariates tested
☐	☒	A description of any assumptions or corrections, such as tests of normality and adjustment for multiple comparisons
☐	☒	A full description of the statistical parameters including central tendency (e.g. means) or other basic estimates (e.g. regression coefficient) AND variation (e.g. standard deviation) or associated estimates of uncertainty (e.g. confidence intervals)
☐	☒	For null hypothesis testing, the test statistic (e.g. F, t, r) with confidence intervals, effect sizes, degrees of freedom and P value noted<br>*Give P values as exact values whenever suitable.*
☒	☐	For Bayesian analysis, information on the choice of priors and Markov chain Monte Carlo settings
☒	☐	For hierarchical and complex designs, identification of the appropriate level for tests and full reporting of outcomes
☒	☐	Estimates of effect sizes (e.g. Cohen's d, Pearson's r), indicating how they were calculated

Our web collection on statistics for biologists contains articles on many of the points above.

Software and code

Policy information about availability of computer code

Data collection	No software was used for data collection.
Data analysis	Illumina Infinium arrays (San Diego, USA; GSAMD-24v2.0, GSA-24v2.0, CoreExome-24v1.1 and CoreExome-24v1.2) were analyzed with GenomeStudio (Illumina).<br>For exome sequencing, raw reads were mapped to the human genome reference sequence (build hg19) with the Novoalign software (V3.08.00, Novocraft Technologies, Selangor, Malaysia). Duplicate reads were then removed using Picard (v. 2.14.0-SNAPSHOT). Base quality score recalibration was performed and variant calling was done with HaplotypeCaller (GATK, v.4.0.3.0).<br>AutoMap is composed of Bash, Perl and R scripts. It was used with BCFTools (v1.9-78-gb7e4ba9), BEDTools (v2.25.0), Perl (v5.22.0), Bash (4.3.48(1)-release) and R (v3.5.1).<br>Probabilities for binomial distributions were calculated with the Perl script written by T.J. Finney (https://www.halotype.com/RKM/figures/TJF/binomial.txt).<br>Overlap of ROHs were obtained with BEDTools (v2.25.0) and the following command: bedtools intersect -a a.bed -b b.bed<br>PLINK (v1.90b5) was used with default parameters for array data:<br>plink --bfile bfile --homozyg --out out --homozyg-window-het 1 --homozyg-density 50 --homozyg-gap 1000 --homozyg-window-missing 5 --homozyg-window-snp 50 --homozyg-snp 100 --homozyg-window-threshold 0.05 --homozyg-kb 1000<br>Samtools (v1.8): samtools mpileup -t DP,AD -ugf ref.fasta input.bam | bcftools call -vmO v -o out.vcf<br>Strelka (v2.9.2): configureStrelkaGermlineWorkflow.py --bam input.bam --referenceFasta ref.fasta --exome<br>HaplotypeCaller: gatk --java-options "-Xmx4g" HaplotypeCaller -R ref.fasta --dbsnp dbsnp.vcf -I $input.bam -O out.vcf<br>AutoMap code is freely available on GitHub (https://github.com/mquinodo/AutoMap/).<br>The tools HomozygosityMapper for WES (no version), HOMWES (genomecombv0.98.8), BCFtools/RoH (v1.9-78-gb7e4ba9), FILTUS (v1.0.5), H3M2 (no version), SavvyHomozygosity (no version), and SavvyVcfHomozygosity (no version) were used with default parameters on exome data.<br>PLINK (v1.90b5) was used on exome data with the following command: plink --bfile bfile --homozyg --out out --homozyg-kb 1000 --homozyg-window-het 3 --homozyg-density 10000 --homozyg-gap 10000 --homozyg-window-missing 10 --homozyg-window-snp 20 --

homozyg-snp 10 --homozyg-window-threshold 0.05 --mac 2
For figures, RStudio (v1.0.153) was used with R (v3.5.1), gridExtra (v2.3) and ggplot2 (v3.3.2).

For manuscripts utilizing custom algorithms or software that are central to the research but not yet described in published literature, software must be made available to editors/reviewers. We strongly encourage code deposition in a community repository (e.g. GitHub). See the Nature Research guidelines for submitting code & software for further information.

Data

Policy information about availability of data

All manuscripts must include a data availability statement. This statement should provide the following information, where applicable:

- Accession codes, unique identifiers, or web links for publicly available datasets
- A list of figures that have associated raw data
- A description of any restrictions on data availability

All data represented in the figures is presented in the Supplementary Table 3

Field-specific reporting

Please select the one below that is the best fit for your research. If you are not sure, read the appropriate sections before making your selection.

[x] Life sciences [ ] Behavioural & social sciences [ ] Ecological, evolutionary & environmental sciences

For a reference copy of the document with all sections, see nature.com/documents/nr-reporting-summary-flat.pdf

Life sciences study design

All studies must disclose on these points even when the disclosure is negative.

Sample size	The training cohort is composed of 26 unrelated individuals. The validation cohort is composed of 26 unrelated individuals. These were all samples available to us and, as written in the text, provided sufficient power to our analysis.
Data exclusions	No data was excluded.
Replication	The validation was performed in 26 independent samples from the training cohort.
Randomization	The 52 samples were splitted into the training and validation cohorts randomly by ethnicity (Portugal or Iran).
Blinding	There was no blinding in the study, since this procedure is not applicable to our analysis.

Reporting for specific materials, systems and methods

We require information from authors about some types of materials, experimental systems and methods used in many studies. Here, indicate whether each material, system or method listed is relevant to your study. If you are not sure if a list item applies to your research, read the appropriate section before selecting a response.

Materials & experimental systems

n/a	Involved in the study	
[x]	[ ]	Antibodies
[x]	[ ]	Eukaryotic cell lines
[x]	[ ]	Palaeontology
[x]	[ ]	Animals and other organisms
[ ]	[x]	Human research participants
[x]	[ ]	Clinical data

Methods

n/a	Involved in the study	
[x]	[ ]	ChIP-seq
[x]	[ ]	Flow cytometry
[x]	[ ]	MRI-based neuroimaging

Human research participants

Policy information about studies involving human research participants

Population characteristics	The participants are coming from families with consanguineous parents either from Portugal or from Iran. The all suffer from inherited vision disorders. Please refer to Supplementary Data file 1 in which we included further details including gender and age.
Recruitment	The participant were recruited in the framework of a project on genetics of diseases of the eye. Selection bias is not applicable to this study as we used all samples available to us.
Ethics oversight	Written informed consent forms were signed by all subjects, recruited at the Ophthalmic Hospital "Dr Gama Pinto" in Lisbon, and at the Fasa and Mashhad Universities of Medical Science in Iran. Ethical approval was obtained from Ethikkommission Nordwest- and Zentralschweiz, Ophthalmic Hospital "Dr Gama Pinto" in Lisbon, and Fasa and Mashhad Universities in Iran.

Note that full information on the approval of the study protocol must also be provided in the manuscript.
